# Carbon Dioxide Laser Microsurgery versus Low-Temperature Plasma Radiofrequency Ablation for T1a Glottic Cancer: A Single-Blind Randomized Clinical Trial

**DOI:** 10.1155/2018/4295960

**Published:** 2018-11-01

**Authors:** Yan Zhang, Binru Wang, Guangbin Sun, Guoliang Zhang, Ling Lu, Gengtian Liang

**Affiliations:** ^1^Department of Pharmacy, The Third Hospital of Wuhan City, Wuhan, Hubei Province, China; ^2^Department of Otolaryngology, The Third Hospital of Wuhan City, Wuhan, Hubei Province, China; ^3^Department of Otolaryngology, Huashan Hospital, Fudan University, Shanghai, China

## Abstract

**Background:**

Very few studies have been conducted to compare carbon dioxide laser microsurgery (CO2-LS) with low-temperature plasma radiofrequency ablation (LTP-RFA) in treating T1a glottic cancer. Therefore, we conducted this study to compare the efficacy of CO2-LS and LTP-RFA to define a superior therapeutic modality for T1a glottic cancer.

**Methods:**

Patients (n=131) with T1a glottic cancer were recruited between January 2010 and September 2014. The included patients were randomly assigned to either receive CO2-LS (n=65) or LTP-RFA (n=66). We conducted the following multidimensional vocal assessments: (i) videostroboscopic evaluation; (ii) auditory-perceptual evaluation; (iii) aerodynamics/ efficiency; (iv) acoustics; and (v) self-assessment questionnaires. Meanwhile, the surgery time and three-year overall survival rates in two groups were recorded. The predefined primary endpoint was overall survival, and the minimum follow-up time was set to six months.

**Results:**

After treatment, we found that the structure and vibration of vocal cord might recover more quickly in patients receiving LTP-RFA than in patients receiving CO2-LS, and moreover, the patients in the LTP-RFA group had the better vocal functions. Meanwhile, the surgery time was significantly less in the LTP-RFA group (8.83±1.59 minutes) than in the CO2-LS group (12.49±1.40 minutes) (p<0.00001). In addition, the two intervention methods had the similar three-year overall survival rates (94% versus 96%, p=0.58).

**Conclusion:**

These results indicated that both LTP-RFA and CO2-LS could effectively treat T1a glottic cancer, and LTP-RFA might have some advantages in voice function. Limited by the relatively small sample size, future studies were needed to validate our conclusion.

## 1. Introduction

Laryngeal squamous cell carcinoma arises from the mucosal surface of the larynx, which is one of the most common head and neck cancers worldwide. In China, the overall incidence rate of laryngeal cancer is about 2.04/100,000, and the male incidence rate is about 3.54/100,000, which is obviously higher than the female incidence rate (about 0.49/100,000) [1]. Along with the enhanced of health awareness and the increased of examination methods, more and more laryngeal cancer patients have been identified at an early stage [2]. Among these patients, glottic cancer is the most common laryngeal cancer. Hoffman et al. reported that there were about 50% of glottis cancer patients among the 158,426 laryngeal cancer patients [3]. Similarly, Ji et al. reported that there were 51.0% of early glottis cancer patients among the 1,115 laryngeal cancer patients [4].

Previous studies reported that the current treatment methods could effectively treat the early stage squamous cell carcinomas of the larynx with high local control rates (about 75%-90%), especially in glottis area [5, 6]. At present, there are three main treatment methods for early glottic cancer: open surgery, radiotherapy, and laser surgery. The aim of the treatment of laryngeal cancer is to provide oncological control and preserve a functioning larynx for patients. Thus, the radiotherapy and laser surgery are the two commonly used methods in clinical practice to treat laryngeal cancer, especially early glottic cancer. In 2006, American Society of Clinical Oncology clinical practice guideline recommended the radiotherapy and laser surgery for T1 glottis cancer and viewed the open surgery as an alternative approach [7]. But many researchers thought that the open surgery could still be one of the first choices for T1 glottis cancer, which was mainly depended on the experience of clinicians and technology adoption [8, 9].

Carbon dioxide laser surgery (CO2-LS) is firstly used to treat T1 glottis cancer in 1975 [10]. This method has many advantages, such as no incision in the neck, high accuracy, minimal injury, quick recovery, and good larynx function preservation. Previous study reported that, after treatment with laser microsurgery, the vocal function of T1a glottic cancer patients gradually improved after about five weeks [11]. Compared to CO2-LS, the low-temperature plasma radiofrequency ablation (LTP-RFA) is a new technology for treating glottic cancer [12, 13]. This method could incise the soft tissue at a lower temperature (40-70°C), provide a clear operative field, and have hemostatic function. However, few studies attempt to compare CO2-LS with LTP-RFA in treating T1a glottic cancer. Therefore, we conducted this study to compare the efficacy of CO2-LS and LTP-RFA to define a superior therapeutic modality for T1a glottic cancer.

## 2. Materials and Methods

### 2.1. Patient Recruitment

This study was reviewed and approved by the Ethics Committees of the Third Hospital of Wuhan City. Patients met the following criteria were recruited: (i) patients with T1a glottic cancer; (ii) patients aged 18 years or older without mental disorders or systemic diseases; (iii) patients were not previously treated for vocal fold or previous cancer; (iv) no neck lymph node metastasis was detected by neck color ultrasonic inspection and enhanced CT (iohexol) before surgery; and (v) both CO2-LS and LTP-RFA were appropriate after a detailed discussion among the clinician, patient and family had occurred. The first patient was recruited in January 2010 and the last patient was recruited in September 2014. All patients were told the purpose of this study and provided the written informed consent before treatment.

### 2.2. Intervention Procedure

After general anesthesia, a pillow was placed under the shoulder of the patients. The adjustable support laryngoscope was imported into the mouth to fully expose the glottis. Then, the surgeon adjusted the microscope to enlarge the surgical field by 20-30 times until he could see it clearly. In the LTP-RFA group, the initial energy level was set to level 7 (ablation) and the coagulation index should be adjusted to level 3 [14]. In the CO2-LS group, to prevent the laser from breaking the balloon and then causing burning, the wet gauze strips were placed on the surface of the tracheal intubation balloon; the CO2 laser was set to a continuous model with a power of 6 to 16W and a spot diameter of 270*μ*m. The tumor was resected along the edge (3-5mm) of the tumor, and the time consumption was recorded. Subsequently, we took a little piece of tissue from the edge of the resected tumor and conducted a quick frozen section. If the result of pathological examination was positive, then we expanded the resection edge and continued to resect the tumor until the result was negative. Patients were given three to five days postoperative antibiotics. Although the surgeon knew the intervention methods that the patients received, the patients, investigator, and data analyst were blind for the intervention methods to avoid potential bias. The devices used in this study included laryngoscope system and operating microscope (Carl Zeiss, Germany); Coblator II plasma surgery system and ReFlex Ultra™ 7070^#^ cutter head (Arthrocare, USA); Sharplan 30C Laser (Lumenis Ltd., Israel).

### 2.3. Postoperative Assessment

The predefined primary endpoint was overall survival (defined as death). The minimum follow-up time was set to six months. According to the European Laryngological Society (ELS) protocol, we conducted the following multidimensional vocal assessments: (i) videostroboscopic evaluation; (ii) auditory-perceptual evaluation; (iii) aerodynamics/efficiency; (iv) acoustics; and (v) self-assessment questionnaires [15]. Five variables (Grade, Breathiness, Asthenia, Roughness, and Strain scale) [16] and three variables (mucosal wave, glottal closure, and symmetry) [17] were used to conduct auditory-perceptual evaluation and videostroboscopic evaluation, respectively. The Multi-Dimensional Voice Program, Model 5150 (KayPENTAX, Lincoln Park, NJ) was used to assess the acoustic parameters (jitter (%), shimmer (%), fundamental, and noise-to-harmonic ratio (NHR)). As an aerodynamic parameter, the maximum phonation time (MPT) was calculated using a sustained vowel/a/phonation. The Voice-Related Quality of Life Measure (V-RQOL) and Chinese version of Voice Handicap Index (VHI) were measured as self-assessment questionnaires.

### 2.4. Statistical Analysis

Data were expressed as mean±standard deviation (SD). Chi-squared test and Student's T test were used when appropriate. Student's T test was used to assess the group differences on aerodynamics/efficiency, acoustics, and self-assessment questionnaires. The repeated measures analysis of variance (ANOVA) was conducted to study the group differences on videostroboscopy and auditory-perceptual evaluation at different time points [18]. The Kaplan-Meier method was used to construct overall survival rate curve. The Cox proportional hazards model was applied to compute relative risk and 95% confidence interval (CI). SPSS 19.0 (SPSS Inc., Chicago, IL, USA) was used, and the significance level was set at p-value<0.05.

## 3. Results

### 3.1. Patient Characteristics

**Figure 1:**
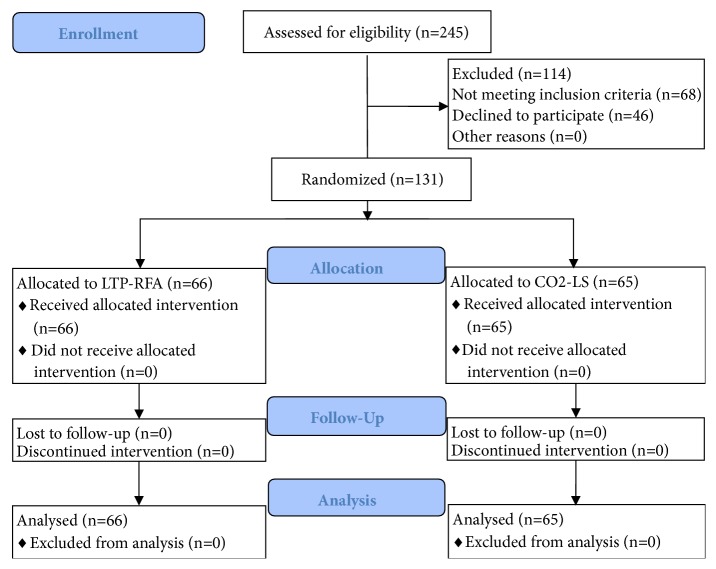
Flow diagram of this study.

**Figure 2:**
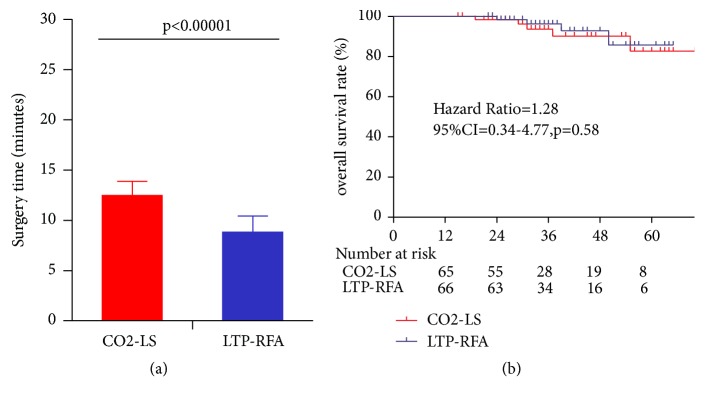
Surgery time and three-year overall survival rates in two groups.

At first, there were 245 candidates, but only 177 patients meeting the abovementioned criteria. Among these patients, 46 patients refused to participate. Finally, the 131 patients with T1a glottic cancer were randomly assigned to receive either CO2-LS (n=65) or LTP-RFA (n=66). We used the data from previous studies to perform sample size estimation [14, 19]. We found that the statistical power could be 0.8 when each group had 60 patients. Thus, this sample size in our study gave us statistical power of 0.83. There were no significant differences in baseline data between the two groups. The detailed information was showed in Table 1. The flow diagram of this study was showed in Figure 1. The type of cordectomy was defined according to the classification of the ELS. In the CO2-LS group, we performed type II cordectomy in 12.3%, type III cordectomy in 80% and type IV cordectomy in 7.7% of patients; in the LTP-RFA group, we performed type II cordectomy in 10.6%, type III cordectomy in 81.8%, and type IV cordectomy in 7.6% of patients. The follow-up periods were similar between the two groups (p=0.43). Compared with the CO2-LS (12.49±1.40 minutes), the LTP-RFA required significantly less surgery time (8.83±1.59 minutes, p<0.00001) (Figure 2(a)). The three-year overall survival rate was 94% in the CO2-LS group and 96% in the LTP-RFA group (p=0.67) (Figure 2(b)).

### 3.2. Videostroboscopic Evaluation

**Figure 3:**
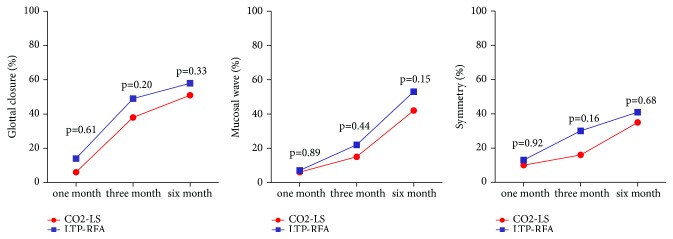
Videostroboscopic evaluations at 1, 3, and 6 months after treatment in two groups.

The videostroboscopic evaluation was conducted at 1, 3, and 6 months after treatment. There were 62 patients in the CO2-LS group and 61 patients in the LTP-RFA group having postoperative vocalizing laryngeal images. The percentages of patients with normal patterns in each of the three variables (glottal closure, mucosal waves, and symmetry) were shown in Figure 3. These results indicated that the structure and vibration of vocal cord might recover more quickly in patients receiving LTP-RFA in comparison with those receiving CO2-LS. In the stroboscopic presentation, about 50% of patients had normal patterns in both the CO2-LS group (glottal closure, 51%; mucosal wave, 41%; symmetry, 35%) and LTP-RFA group (glottal closure, 67%; mucosal wave, 60%; symmetry, 41%) at 6 months after treatment.

### 3.3. Auditory-Perceptual Evaluation

**Figure 4:**
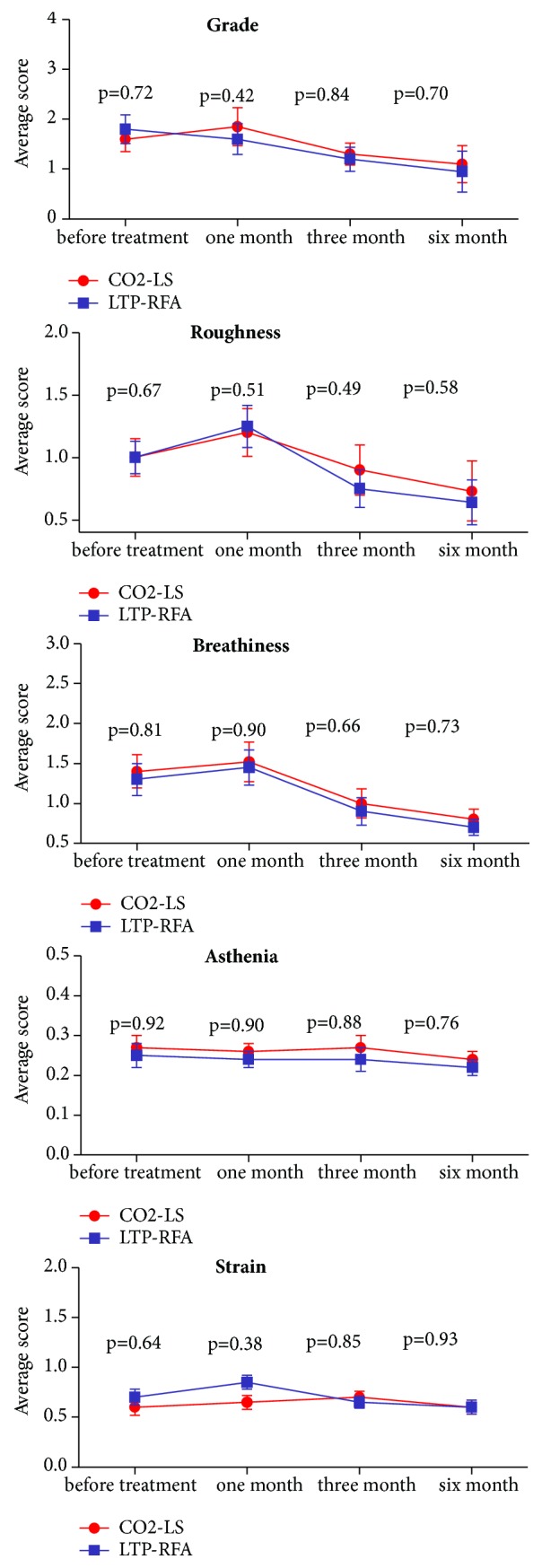
Auditory-perceptual evaluations before treatment and at 1, 3, and 6 months after treatment in two groups.

The auditory-perceptual evaluation was conducted before treatment and at 1, 3, and 6 months after treatment. At each time point, the two groups had similar scores of grade, roughness, breathiness, asthenia, and strain scale (Figure 4). At 3 months after treatment, we observed somewhat better scores of grade and breathiness in both the CO2-LS group (Grade=1.3, Breathiness=1.0) and LTP-RFA group (Grade=1.2, Breathiness=0.9) compared with their pretherapeutic scores (CO2-LS group, Grade=1.6, Breathiness=1.4; LTP-RFA group, Grade=1.8, Breathiness=1.3). Meanwhile, we also observed a slight deterioration and subsequent recovery of roughness in both groups during the first three months treatment. These two intervention methods had little effect on the scores of both asthenia and strain.

### 3.4. Aerodynamics and Self-Assessment Questionnaires

**Figure 5:**
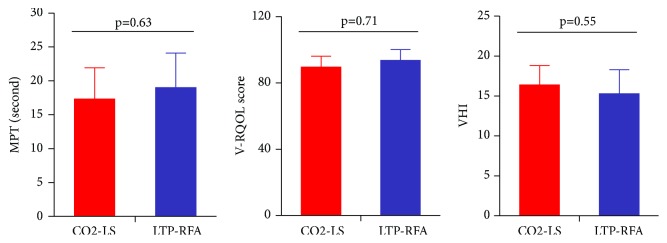
Aerodynamics and Self-Assessment questionnaires at 6 months after treatment in two groups.

The MPT, V-RQOL, and VHI were recorded at the time point of six months after treatment. As shown in Figure 5, compared with the CO2-LS group (17.3±4.6 seconds), the LTP-RFA group had a nonsignificantly higher MPT (19.0±5.1 seconds) (p=0.63). We also did not observe any significant differences on the V-RQOL (89.6±6.5 versus 93.5±6.9, p=0.71) and VHI (16.4±2.4 versus 15.3±3.0, p=0.55) between the CO2-LS group and LTP-RFA group.

### 3.5. Acoustics

**Figure 6:**
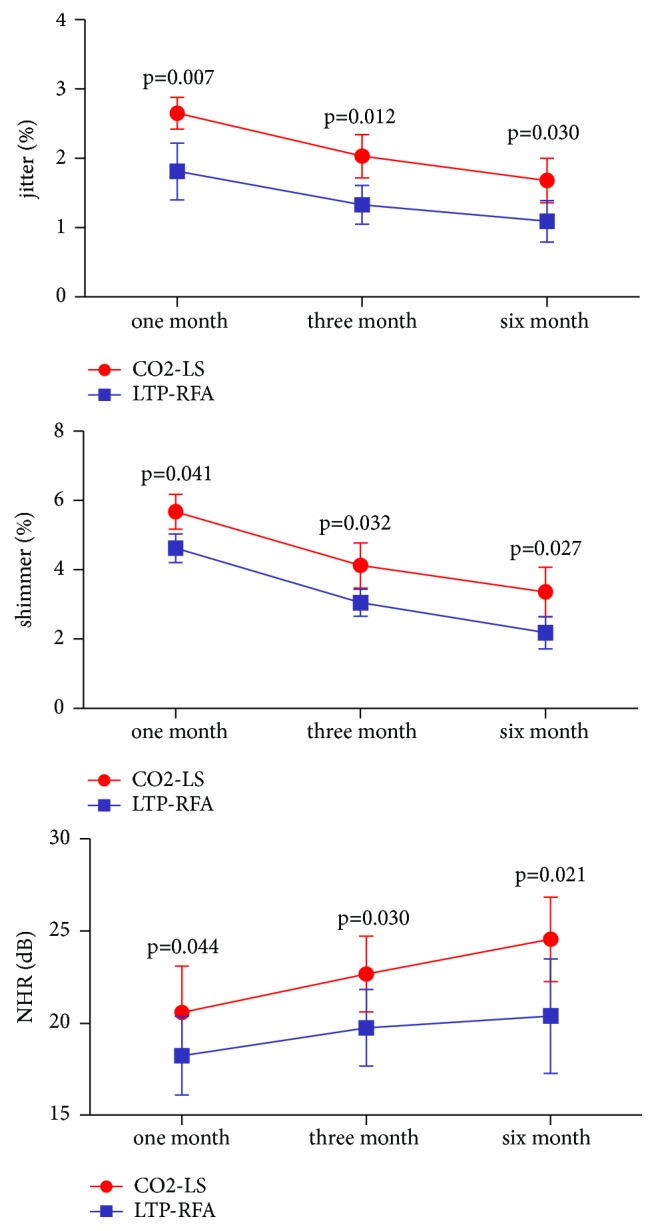
Acoustic parameters at 1, 3, and 6 months after treatment in two groups.

These acoustic parameters were recorded at 1, 3, and 6 months after treatment. As shown in Figure 6, the three variables (jitter (%), shimmer (%), and NHR (dB)) were significantly improved over time in both groups. The significant effect of time (p<0.00001) indicated that both CO2-LS and LTP-RFA could effectively improve the three variables. Meanwhile, the significant effect of group x time interaction (p=0.025) indicated that the improvements were significantly different between the two groups. At each time point, compared with the CO2-LS group, the LTP-RFA group had the significantly lower average jitter (%), shimmer (%), and NHR (dB) scores. These results showed that the LTP-RFA could produce the significantly better vocal functions than the CO2-LS.

## 4. Discussion

This randomized controlled trial was conducted to compare CO2-LS with LTP-RFA in treating T1a glottic cancer. Considering the equivalent local control rate of these two intervention methods, the posttherapeutic laryngeal function was viewed as the critical role in determining the superior therapeutic modality for early glottic cancer. Up to now, few studies have been conducted to simultaneously perform the subjective and objective evaluation of vocal function according to multidimensional analyses. Here, we found that, compared to the CO2-LS, the LTP-RFA needed significantly less surgery time, which might make LTP-RFA more suitable for debilitated or elderly patients. These two intervention methods had the similar three-year overall survival rates. Meanwhile, using multidimensional analyses, we found that the structure and vibration of vocal cord might recover more quickly in patients receiving LTP-RFA than in patients receiving CO2-LS, and moreover, the patients in the LTP-RFA group had the significantly better vocal functions. These results showed that the LTP-RFA had some advantages over CO2-LS in treating T1a glottic cancer.

These two treatment modalities have their own advantages and disadvantages. The cutter head of LTP-RFA system could bend in a wide range, which makes it easier for the surgeon to adjust the angle during the surgery. Thus, using LTP-RFA is more easily to conduct the resection or ablation of cancer tissue in hidden parts [20]. Moreover, compared to the CO2-LS system, the LTP-RFA system is relatively cheaper and easily to perform clinically [14]. However, the thermal efficiency of plasma radiofrequency is relatively low, which makes LTP-RFA have certain limitations in attaining intraoperative hemostasis [19]. In the case of arterial hemorrhage, it is sometimes necessary to use a high frequency electric knife to stop the bleeding [19]. Although the LTP-RFA has no risk of airway burns, its relatively thick cutter head (about 5.0mm in diameter) usually is relatively difficult to confirm the safety margin, while the CO2-LS has the characteristics of precision cutting, because of the only 270*μ*m spot diameter of CO2 laser. In addition, Aaltonen et al. reported that the overall voice quality was similar between laser surgery group and radiation therapy group [21]. These results indicated that the surgeon should take both patient-related factors and advantages/disadvantages of treatment modalities into consideration when choosing a treatment option.

Some studies reported that both radiotherapy and laser surgery could provide the high probability of successful local control (about 90% five-year overall survival rate) for early glottic cancer [22–24]. Meanwhile, several cross-sectional studies were conducted to explore the advantages of radiotherapy and laser surgery by examining the posttherapeutic vocal outcomes of early glottic cancer patients [25–28]. The better posttherapeutic vocal quality was found in radiotherapy group in some studies [25, 26], but also in laser surgery group in other studies [27, 28]. Nevertheless, only one or two aspects of vocal function were assessed in these studies, whereas the ELS insisted that it was very important to adequately describe the vocal function by multidimensional assessments of vocal pathology [14]. According to the ELS guideline, a recent study reported that early glottis cancer could be successfully treated by either radiotherapy or laser therapy (excision with focused mode using lower power) with equivalent posttherapeutic laryngeal function and quality of life (QoL) [29]. These studies demonstrated that which one treatment modality was better in treating early glottic cancer still remained controversial and was needed high-quality randomized controlled trial to find out. Here, we found the better posttherapeutic laryngeal function in the LTP-RFA group than in the CO2-LS group. Our high-quality randomized trial might make our conclusion more robust.

Nevertheless, our study inevitably had several limitations. Firstly, the sample size was relatively small in each group; then future studies with a large cohort of patients were needed to validate our conclusion. Secondly, all patients with T1a glottic cancer were recruited from the same city and were of the same ethnicity; this point might limit the applicability of our conclusion [30–32]. Thirdly, the follow-up period was relatively short (median, 33 months in the CO2-LS group, 36 months in the LTP-RFA group), which made it impossible to compare the five-year overall survival rates of these two intervention methods. Future studies with long-term follow-up periods were needed to further investigate the efficacy and acceptability of these two treatment modalities. Fourthly, we did not compare the cumulative costs of these two treatment methods; future studies should take cumulative cost into consideration when comparing the efficacy of these two treatment methods. Finally, not all patients received the same type cordectomy, which suggested that the obtained different results might also be associated with the entity of the excision and not only the intrinsic differences among the two devices used. Future studies should explore whether or not the results were similar or not when all patients received the same type cordectomy.

In conclusion, this randomized controlled trial showed that the surgery time was significantly less in the LTP-RFA group than in the CO2-LS group. These two intervention methods had the similar three-year overall survival rates. Meanwhile, the structure and vibration of vocal cord might recover more quickly in patients receiving LTP-RFA than in patients receiving CO2-LS, and moreover, the patients in the LTP-RFA group had the significantly better vocal functions. These results indicated that the LTP-RFA might have some advantages over CO2-LS in treating T1a glottic cancer. However, limited by the relatively small sample sizes, these results needed future studies to further validate. The original data could be found in Supplementary File 1.

## Figures and Tables

**Table 1:** Baseline characteristics of patients with T1a glottic cancer.

**Variables**	**CO2-LS**	**LTP-RFA**	**p-value**
n	65	66	-
Age, year, mean (SD)	56.71 (9.90)	57.15 (10.5)	0.82
Female/Male	55/10	53/13	0.52
BMI, kg/m^2^	23.98 (3.77)	23.61 (4.03)	0.40
Smoking, Yes/No	22/43	29/37	0.24
Alcohol abuse, Yes/No	11/54	9/57	0.60
Surgery time, minute, mean (SD)	12.49 (1.40)	8.83 (1.59)	<0.00001
Follow-up, mouth, mean (range)	33 (15-70)	36 (22-65)	0.43

## Data Availability

The supplementary material is the original data of this study.

## Supplementary Materials

Supplementary Materials The supplementary file is the "original data for the results of this study".

## References

[1] Ling-Bin D., Wei-Min M., Wan-Qing C. (2012). Incidence and mortality of larynx cancer in China dudng 2003-2007. *Chinese Journal of Epidemiology*.

[2] Mostafa B. E., Shafik A. G., Fawaz S. (2007). The role of flexible autofluorescence laryngoscopy in the diagnosis of malignant lesions of the larynx. *Acta Oto-Laryngologica*.

[3] Hoffman H., Porter K., Karnel L. (2006). Laryngeal cancer in united states: changes in demographics, patterns of care, and survival. *Laryngoscope*.

[4] Wen-Yue J., Qing D., Chao G., Dian-Ge W. (2004). Survival analysis of 1115 patients with laryngeal carcinoma. *Chinese Journal of Ophthalmology and Otorhinolaryngology*.

[5] O'Hara J., Markey A., Homer J. J. (2013). Transoral laser surgery versus radiotherapy for tumour stage 1a or 1b glottic squamous cell carcinoma: Systematic review of local control outcomes. *The Journal of Laryngology & Otology*.

[6] Van Loon Y., Sjögren E. V., Langeveld T. P. M., Baatenburg De Jong R. J., Schoones J. W., Van Rossum M. A. (2012). Functional outcomes after radiotherapy or laser surgery in early glottic carcinoma: A systematic review. *Head & Neck*.

[7] Pfister D., Lauris S., Weinstein G. (2006). American society of clinical oncology clinical practice guideline for the use of larynx-preservation strategies in the treatment of laryngeal cancer. *Journal of Clinical Oncology*.

[8] Silver C. E., Beitler J. J., Shaha A. R., Rinaldo A., Ferlito A. (2009). Current trends in initial management of laryngeal cancer: the declining use of open surgery. *European Archives of Oto-Rhino-Laryngology*.

[9] Mendenhall W. M., Werning J. W., Hinerman R. W., Amdur R. J., Villaret D. B. (2004). Management of T1-T2 Glottic Carcinomas. *Cancer*.

[10] Strong M. S. (1975). Laser excision of carcinoma of the larynx. *The Laryngoscope*.

[11] Brøndbo K., Benninger M. S. (2004). Laser resection of T1a glottic carcinomas: Results and postoperative voice quality. *Acta Oto-Laryngologica*.

[12] Timms M. S., Bruce I. A., Patel N. K. (2007). Radiofrequency ablation (coblation): a promising new technique for laryngeal papillomata. *The Journal of Laryngology & Otology*.

[13] Carney A. S., Evans A. S., Mirza S., Psaltis A. (2010). Radiofrequency coblation for treatment of advanced laryngotracheal recurrent respiratory papillomatosis. *The Journal of Laryngology & Otology*.

[14] Shuang Y., Chao L., Yongwang H., Shuang W. (2015). Comparison between Radiofrequency Coblation and CO2 Laser for the Treatment of Early Glottic Carcinoma. *Journal of Audiology and Speech Pathology*.

[15] Dejonckere P. H., Bradley P., Clemente P. (2001). A basic protocol for functional assessment of voice pathology, especially for investigating the efficacy of (phonosurgical) treatments and evaluating new assessment techniques: guideline elaborated by the Committee on Phoniatrics of the European Laryngological Society (ELS). *European Archives of Oto-Rhino-Laryngology*.

[16] Isshiki N., Okamura H., Tanabe M., Morimoto M. (1969). Differential diagnosis of hoarseness. *Folia Phoniatrica et Logopaedica*.

[17] Bless D. M., Hirano M., Feder R. J. (1987). Videostroboscopic evaluation of the larynx.. *Ear, Nose & Throat Journal*.

[18] Chen J.-W., Xie S.-Q. (2018). Agomelatine versus paroxetine in treating depressive and anxiety symptoms in patients with chronic kidney disease. *Neuropsychiatric Disease and Treatment*.

[19] Zhang Q.-F., Liu D.-L., Zhang Y. (2011). Preliminary investigation of coblation for early glottic carcinoma. *Chinese Journal of Otorhinolaryngology Head and Neck Surgery*.

[20] Pradhan S. A., Pai P. S., Neeli S. I., D'Cruz A. K. (2003). Transoral laser surgery for early glottic cancers. *Archives of Otolaryngology—Head and Neck Surgery*.

[21] Aaltonen L.-M., Rautiainen N., Sellman J. (2014). Voice quality after treatment of early vocal cord cancer: a randomized trial comparing laser surgery with radiation therapy. *International Journal of Radiation Oncology • Biology • Physics*.

[22] Sigston E., De Mones E., Babin E. (2006). Early-stage glottic cancer: Oncological results and margins in laser cordectomy. *Archives of Otolaryngology—Head and Neck Surgery*.

[23] Sjögren E. V., Langeveld T. P. M., De Jong R. J. B. (2008). Clinical outcome of T1 glottic carcinoma since the introduction of endoscopic CO2 laser surgery as treatment option. *Head & Neck*.

[24] Sjögren E. V., Wiggenraad R. G. J., Le Cessie S., Snijder S., Pomp J., De Jong R. J. B. (2009). Outcome of radiotherapy in T1 glottic carcinoma: A population-based study. *European Archives of Oto-Rhino-Laryngology*.

[25] Rydell R., Schalén L., Fex S., Elner Å. (1995). Voice evaluation before and after laser excision vs. Radiotherapy of t1a glottic carcinoma. *Acta Oto-Laryngologica*.

[26] Epstein B. E., Lee D.-J., Kashima H., Johns M. E. (1990). Stage T1 glottic carcinoma: Results of radiation therapy or laser excision. *Radiology*.

[27] Rosier J.-F., Grégoire V., Counoy H. (1998). Comparison of external radiotherapy, laser microsurgery and partial laryngectomy for the treatment of T1N0M0 glottic carcinomas: A retrospective evaluation. *Radiotherapy & Oncology*.

[28] Wedman J., Heimdal J.-H., Elstad I., Olofsson J. (2002). Voice results in patients with T1a glottic cancer treated by radiotherapy or endoscopic measures. *European Archives of Oto-Rhino-Laryngology*.

[29] Kono T., Saito K., Yabe H., Uno K., Ogawa K. (2016). Comparative multidimensional assessment of laryngeal function and quality of life after radiotherapy and laser surgery for early glottic cancer. *Head & Neck*.

[30] Chen J., Bai S., Li W. Urinary biomarker panel for diagnosing patients with depression and anxiety disorders. *Translational Psychiatry*.

[31] Lin L., Chen X.-M., Liu R.-H. (2017). Novel urinary metabolite signature for diagnosing postpartum depression. *Neuropsychiatric Disease and Treatment*.

[32] Zheng P., Chen J.-J., Zhou C.-J. (2016). Identification of sex-specific urinary biomarkers for major depressive disorder by combined application of NMR- and GC-MS-based metabonomics. *Translational Psychiatry*.

